# Combined Effects of Virtual Reality and Computer Game-Based Cognitive Therapy on the Development of Visual-Motor Integration in Children with Intellectual Disabilities: A Pilot Study

**DOI:** 10.1155/2021/6696779

**Published:** 2021-07-05

**Authors:** Si-nae Ahn

**Affiliations:** Department of Occupational Therapy, Cheongju University, Cheongju, Republic of Korea

## Abstract

**Purpose:**

Visual-motor integration is a good indicator of a child's overall developmental and functional level. This study investigated the combined effects of virtual reality (VR) and computer game-based cognitive therapy on the development of visual-motor integration in children with intellectual disabilities.

**Methods:**

The study used a single-group pre-post study design and 13 children with intellectual disabilities who were recruited from a community rehabilitation center participated in the study. We used the Wii VR video game and the CoTras computer game to deliver cognitive therapy over 12 sessions. The Bruininks-Oseretsky Test of Motor Proficiency-2 (BOT-2) was used to evaluate motor function related to visual-motor integration to identify changes in function, and the Developmental Test of Visual Perception-2 (DTVP-2) was used to assess changes in visual perception function associated with visual-motor integration.

**Results:**

The VR and computer game-based cognitive therapy has shown significant difference in total standard score of BOT-2 associated with visual-motor integration representing improved motor function (*p* < 0.01). Comparison of the DTVP-2 scores showed the significant difference in visual-motor integration of spatial relation and visual-motor speed (*p* < 0.05), motor-reduced visual perception (*p* < 0.01), and general visual perception (*p* < 0.01).

**Conclusions:**

Results of this study provide useful evidence supporting the possibility of combined VR and computer game-based cognitive therapy for children with intellectual disabilities.

## 1. Introduction

Neurodevelopmental disorders are classified into ten categories: intellectual disabilities, autism spectrum disorder (ASD), attention-deficit/hyperactivity disorder, global developmental delay, communication disorders, specific learning disorders, developmental coordination disorders, stereotypical movement disorders, tic disorders, and Tourette's syndrome. Among these disorders, the highest rates are seen for intellectual disability and ASD. In particular, intellectual disability is most often seen in combination with other disabilities and affects many other disabilities according to the severity of the intellectual disability. Intellectual disability is characterized by limitations in both intellectual functioning and adaptive behavior and, by definition, manifest before 18 years of age [1, 2].

The *Diagnostic and Statistical Manual of Mental Disorders*, *Fifth Edition*, defines intellectual disability as deficits in verbal comprehension, working memory, perceptual reasoning, and cognitive efficacy [1]. Previous studies have shown that intellectual disabilities manifest through deficits in working memory [3], perceptual performance [4, 5], and fine and gross motor skills [6]. Intellectual disability has a limitation in the process of development, in which there are challenges in visual-motor integration and conceptual, social, and practical life skills, and there is a need for intermittent support in certain life activities [7]. Visual-motor integration in individuals with intellectual disability is particularly important because this skill deficit affects the developmental tasks of cognition, self-care, education, and participation in school functions; therefore, it is an important prerequisite for independence in the activities of daily living.

Visual-motor integration refers to the coordination of fine motor and visual perceptual skills and is a good indicator of a child's overall level of functioning as these skills correlate significantly with academic achievement and intellectual functioning. Previous studies have used measures of intellectual functioning to predict visual-motor integration skills. Wuang et al. reported that processing speed, verbal comprehension, and perceptual organization predict the integration of visual perception and motor function [8]. Others have demonstrated that intelligence quotient (IQ) scores within the Wechsler Intelligence Scales are strongly correlated with visual-motor integration [4, 9].

In children, visual-motor integration is dependent on intact visual perception, fine motor coordination, motor inhibition, and sustained attention [10]. Evaluating visual-motor integration reflects an essential element of an individual child's participation in school functions and daily intellectual interactions [11]. Particularly for children with intellectual disabilities, it can provide a safe setting in which to practice skills that may otherwise carry too many perceived risks in the real world. Virtual technology has been used for the acquisition of skills necessary for visual perception, motor skills, and school functions [12]. Visual-motor integration has been studied widely in clinical populations and, considering the importance of the relationship between visual-motor integration and intellectual functioning, we hypothesized that deficits in visual-motor integration affect intellectual disabilities in children.

Children with intellectual disabilities often reject real experiences. In adulthood, acquiring or maintaining these skills through practice is difficult for the same reason. Virtual environments play a role in rehabilitation, especially in the acquisition and maintenance of skills necessary for independent living. For people with intellectual disabilities, virtual environments can contribute to the process of intervention in rehabilitation [12]. In many fields of pediatric research, computer game-based interventions are being used to improve visual perception, motor skills, emotion, language, and social skills [13–16]. In addition, computer game-based interventions provide a safe and nonthreatening context for practicing and acquiring new and difficult skills [17]. In particular, the most common computer game-based intervention is computer game-based cognitive therapy. The advantages of the computerized cognitive rehabilitation program are subdivided by cognitive domain, and economic benefits and immediate feedback are possible by controlling the flexibility of treatment and shortening the treatment time, and it has the advantage that the difficulty can be adjusted according to the level [18].

Virtual reality (VR) enables manipulation of the environment and may stimulate visual perceptional activation and motivation in children with intellectual disabilities, enabling them to learn and to better manage their difficulties [19]. For these reasons, VR can enable the rehabilitation in an ecologically valid environment and possesses many qualities making it appropriate for use in rehabilitative interventions. The ecological validity of VR derives from the precise presentation and control of dynamic perceptual stimuli. Another important added value of VR is the presence that individuals can experience in an immersive VR environment [20]. The VR ranges from nonimmersive to fully immersive. There are different types of VR, including immersive virtual reality, desktop virtual reality, projection virtual reality, and simulation virtual reality [21]. A previous study did not find a significant difference between the performances of immersed and nonimmersed participants [12]. However, a previous study has suggested that individuals with high presence, whether immersive or nonimmersive, have achieved better overall performance [12]. Moreover, until recently, the downside of VR was its high cost and hardware limitations [20]. Commercial nonimmersive VR products offer motion-based gaming VR at an affordable price and can be easily purchased even at home. For the use of nonimmersive VR in rehabilitation to be useful, it requires individualizing treatment and tailoring the intervention to each individual's needs. In this study, in order to supplement the limitations of VR, the purpose of this study was to investigate the impact of providing individualized computer-based cognitive therapy along with nonimmersive VR for children with intellectual disabilities.

Motor learning studies show that positive transfer occurs when previous experiences promote the learning of a new skill or the performance of a skill in a new context. The transfer-appropriate processing theory is important in the similarity of the cognitive processes of learning or performance required in the performance situations. The more similar the performance components or context is, the stronger the amount of positive transfer. This suggests that the positive transfer is due to the similarity of cognitive processing characteristics required in a performance components or context [22]. This study is aimed at evaluating the applicability of interventions that promote visual motor integration by combining VR and computer game-based cognitive therapy performed in similar technologies and contexts to evoke these positive transitions in children with intellectual disabilities.

Nevertheless, there appears to be a lack of clinical evidence that children with intellectual disabilities can benefit from the combined VR and computer game-based cognitive therapy. This pilot study is aimed at examining changes in visual-motor integration of children with intellectual disabilities and exploring the feasibility of combined VR and computer game-based cognitive therapy in this population. The specific purpose was to investigate the effects of combined VR and computer game-based cognitive therapy on the development of visual-motor integration in children with intellectual disabilities.

## 2. Materials and Methods

### 2.1. Study Design

This study used a single-group pre-post study design. The study was approved by the C University Institutional Review Board (10141107-201808-HR-015-02).

### 2.2. Participants

Children eligible to participate met the following inclusion criteria: age, 7 to 13 years; IQ score range, 55 to 85; desired goal of visual motor integration by parents; children who can play Wii console games and computer games for 20 minutes in a sitting or standing position; children who can press a button and hold a Wii console remote control for 20 minutes; children who can follow the therapist's verbal instructions; and agreement to participate in the study. Fifteen participants were recruited, but two were drop out from the evaluation session. Finally, thirteen participants participated in this study. The following exclusion were above 14 years, normal IQ score range, and disagreement to participate in the study.

### 2.3. Procedures

**Figure 1:**
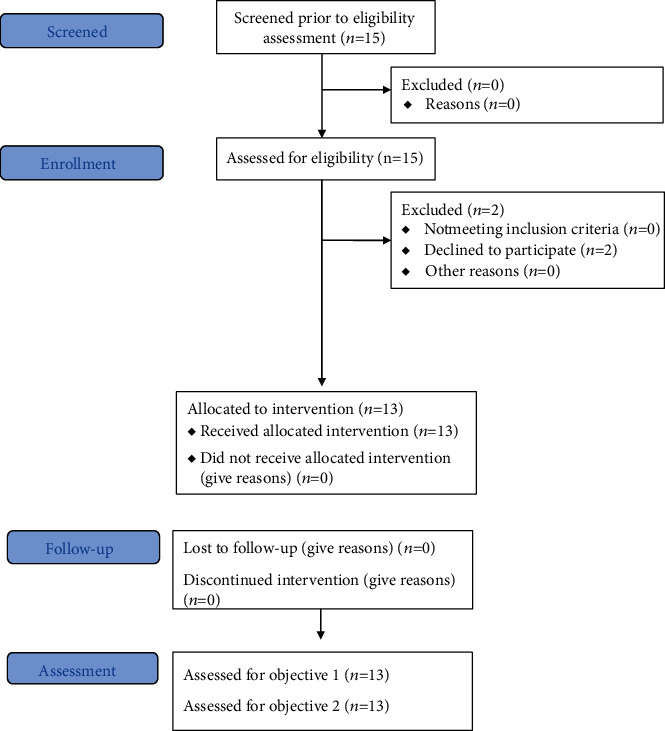
Study process.

The children were recruited by a researcher according to inclusion criteria. In the intervention period, the children underwent VR and computer game-based cognitive therapy over 12 sessions for forty minutes once a week. In the assessment period, the pre- and post-intervention measurements of visual visual-motor integration were taken in the first and last sessions, respectively. To assess the changes in visual-motor integration, the Bruininks-Oseretsky Test of Motor Proficiency, 2nd Edition (BOT-2) and the Developmental Test of Visual Perception, 2nd Edition (DTVP-2) were used. The BOT-2 was used to evaluate motor function related to visual-motor integration to identify changes in function and the DTVP-2 was used to assess changes in visual perception function associated with visual-motor integration (Figure 1).

### 2.4. Intervention

**Figure 2:**
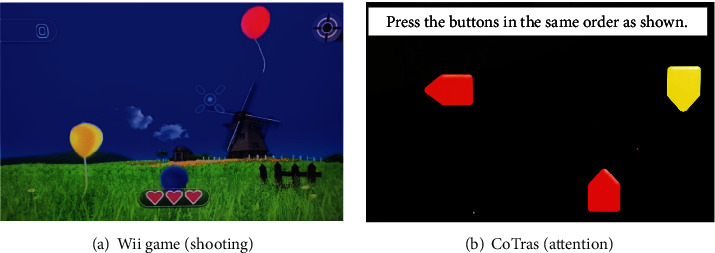
Examples of Wii game and CoTras screens.

All children in the study received conventional therapy and VR and computer game-based cognitive therapy. The VR and computer game-based cognitive therapy involved an occupational therapist who met regularly to plan the child's goals and progress. Our study used Wii console VR video game as a VR game and CoTras program as a computer game-based cognitive therapy for children with intellectual disabilities. Commercial VR products are easy to purchase because they provide VR for motion-based games at an affordable price and have high accessibility at children's homes or rehabilitation centers. The Wii console VR video game (Nintendo Co. Ltd., Kyoto, Japan) a representative motion-based game VR was used in this study. The Wii console VR video game enables participation of individuals whose functional limitations otherwise prevent real-life practice of the sporting activities [23]. We selected the Wii program for several reasons, particularly because it was the most appropriate option for our participants in terms of functional hand skills to manage the Wii remote console, gross motor skills to work in either a sitting or standing position, and visual perception and cognitive skills necessary to understand the games, follow directions, and stay on task [24]. For this study, we used the Wii video game tailored to users with intellectual disabilities. All participants started the same stage from step 1 and performed four sports games in each session. The four video games included shooting, jumping, whack a mole and seesaw, and the duration of the four sports games was 20 minutes (Figure 2).

The CoTras is a computerized cognitive rehabilitation program that has already proven its effectiveness in many studies in Korea. The CoTras program consists of approximately 4,000 computer game-based training tasks in skills including coordination, attention, visual perception, visual motor organization, memory, and orientation. The CoTras is easy to train using the touch monitor and CoTras controller with a child-friendly character design and voice. The difficulty level of the tasks can be modified to suit the individual participant's abilities. Training data are automatically stored, enabling development of an appropriate treatment plan [25]. Participants received five visual perception training sessions involving spatial relations, spatial memory, concentration, eye-hand coordination, eye movement, and figure-ground perception (Figure 2). The five CoTras training session was performed for 20 min. Therefore, all participants received the Wii and CoTras intervention for 40 min per session.

### 2.5. Assessment of Visual-Motor Integration

Visual-motor integration scores from the BOT-2 and DTVP-2 were recorded to determine the impact of the VR and computer game-based cognitive therapy on children's visual-motor integration. The BOT-2 was used to evaluate the impact on motor function related to visual-motor integration. The BOT-2 is a validated tool to identify fine and gross movement skills assessment designed to be used to evaluated people between the ages of 4 and 21 years [26]. The BOT-2 shows different reference values for boys and girls in the field of motor accuracy and visual-motor integration [27]. The testing battery measures fine and gross movement skills using 53 test items in eight subtests: fine motor precision (seven items), fine motor integration (seven items), manual dexterity (five items), bilateral coordination (eight items), balance (nine items), running speed and agility (five items), upper limb coordination (seven items), and strength (five items). The reliability of the BOT-2 subitem is 0.70 to 0.80, the domain reliability coefficient is 0.80 to 0.90, and the reliability of the entire items is 0.90 [26]. In this study, we calculated a total standard score and percentile rank based on all subtests.

Assessments were conducted to identify changes in visual perception and motor functions related to visual-motor integration. The DTVP-2 was used to specifically assess changes in visual perception function associated with visual motor integration. The DTVP-2 measures visual-motor integration and visual perception in children aged 4 to 10 years [28]. The DTVP-2 is a validated tool that assesses visual perception through eight perception-related motor function subtests: eye-hand coordination, position in space, copying, figure-ground perception, spatial relation, visual closure, visual-motor speed, and form constancy. Four visual-motor integration items (eye-hand coordination, shape copying, spatial relationship, and visual motion speed) and four motor-reduced visual perception items (position in space, figure ground, visual closure, and form constancy) are final scores. Each subtest consists of items of increasing difficulty, and testing is discontinued based on stop rules in each subtest. The DTVP-2 is frequently used by occupational therapists to evaluate school-aged children. The reliability of the DTVP-2 subitem is 0.83 to 0.95, the composite reliability coefficient is 0.94 to 0.97, and the reliability of the composite of general visual perception is 0.97 [29]. In this study, scores were calculated for general visual perception, visual motion integration, and motor-reduced visual perception, and analysis was performed for each detailed visual perception areas.

### 2.6. Data Analysis

Descriptive statistics and frequency analysis were performed on the general characteristics of the study participants. The Wilcoxon signed-rank test was used to find significant differences between pretest and posttest within the group. To indicate statistical significance, a *p* value of <0.05 was used. All analyses were performed using SPSS version 18.0 (IBM Corporation, Armonk, NY, USA). In addition to performing statistical analysis and validation of significance for all comparisons, the effect size was recorded to indicate how important the difference was. Effect sizes of the intervention, as measured by rank-biserial correlation, were calculated by Spearman's correlation coefficient from the *p* value of the pretest and posttest scores [30]. In the interpretation of Spearman's correlation coefficient, above 0.7 was strong and moderate was 0.4 to 0.6 [31].

## 3. Results

### 3.1. Characteristics of Participants

A total of 13 children, with intellectual disability, participated in the present study. Seven boys (53.8%) and six girls (46.2%), with an average chronological age of 9.2 ± 1.7 years, all with the dominant right hand, completed the study. At the level of motor function, eight children (61.5%) were rated well-below average and five children (38.5%) were below average. At the level of general visual perception, four children (30.8%) were rated very poor, one child (7.7%) was poor, three children (23.1%) were below average, three children (23.1%) were average, and two children (15.4%) above average (Table 1).

### 3.2. Changes in Visual-Motor Integration

The changes in the children's levels of visual-motor integration, as assessed by differences pre- and post-intervention scores, are presented in Table 2. The children's visual motor integration scores were evaluated by dividing into BOT-2, which reflects motor function, and DTVP-2, which reflects visual perception. A Wilcoxon signed-rank test showed a significant improvement in total standard score BOT-2 between pretest (*M* = 27.15, SD = 7.55) and posttest (*M* = 35.23, SD = 13.09). The positive correlation was strong (*r* = 0.744). For the percentile rank BOT-2, there was a significant improvement between pretest (*M* = 3.46, SD = 4.99) and posttest (*M* = 19.61, SD = 26.87). The positive correlation was moderate (*r* = 0.631).

For the general visual perception in DTVP-2, a significant improvement between pretest (*M* = 97.76, SD = 23.21) and posttest (*M* = 105.92, SD = 28.13) (*p* < 0.01) and the positive correlation was strong (*r* = 0.893). Also, in the motor-reduced visual perception in DTVP-2, there was a significant improvement between pretest (*M* = 109.23, SD = 23.50) and posttest (*M* = 120.61, SD = 27.57) (*p* < 0.01), and the positive correlation was strong (*r* = 0.942). In the subtest of motor-reduced visual perception, only visual closure subtest was not significant (*p* > 0.05). The differences in visual motor integration of the DTVP-2 of pretest (*M* = 86.46, SD = 22.42) did not significantly differ from those of posttest (*M* = 90.46, SD = 27.07, *p* = 0.182), but the positive correlation was strong (r = 0.908). In the subtest of visual motor integration, spatial relation and visual motor speed subtest exhibited strong correlations (*r* = 0.920, 0.723).

## 4. Discussion

The aim of this study was to assess the changes in visual-motor integration of children with intellectual disabilities following a combined VR and computer-based cognitive therapy. Visual-motor integration skills are dependent on intact visual-perception, sustained attention, fine motor coordination, and motor inhibition [32]. In this study, the evaluation of visual motor integration was divided into two types of assessment. Visual motor integration that emphasized motor function was evaluated as BOT-2, and visual motor integration that emphasized visual perception function was evaluated as DTVP-2. By recording and analyzing scores before and after the intervention in two assessments, this study identified the impact of VR and computer game-based cognitive therapy on visual motor integration in children with intellectual disabilities.

In this study, the BOT-2 test was used to assess visual motor integration based on motor skills. The pre- and postintervention comparison showed that a significant improvement in the total score. On the other hand, DTVP-2 showed that there was a significant improvement in visual motor integration, and the general visual perception has also improved significantly. Specifically, only the visual motor speed item was improved among the four subtests related to visual motor integration. For the remaining three items including visual motor index, eye-hand coordination, shape copying, and spatial relationship improved after treatment compared to before treatment, but there was no significant difference and the effect size was small, while the motor-reduced visual perception index was significantly improved and the effect size was medium to large.

Visual-motor integration (VMI) is defined as fine motor and coordination of visual perception [33]. All children in this study had lower than average motor skills. Improvements in visual motor integration, assessed based on motor function, are thought to have a greater impact on lower motor function in children. For this reason, it is proposed that the improvement of visuomotor control by VR and computer-based cognitive therapy occurs at a higher level in terms of the efficiency of sensorimotor systems in processing visual information for motor control. Since this study provided both virtual reality therapy and computer-based cognitive therapy, it is thought that it was an intervention method that could further compensate for the cognitive deficits of children with intellectual disabilities. According to results of this study, VR and computer game-based cognitive therapy is an intervention method for children with intellectual disabilities, with moderate to strong impacts on visual perception and motor functions.

A particular advantage of using VR and computer games as cognitive therapy is that they are free from the social demands that are often challenging and confusing for children with intellectual disabilities. In contrast to traditional social environments, VR and computer-based cognitive therapy can provide immediate, predictable, and repeatable responses [34]. Children with intellectual disabilities are often denied real-world experiences, which for normally developed children provide the opportunity to acquire skills in developmental processes [35, 36]. Therefore, it is necessary to provide these children with an opportunity to acquire these skills through alternative means such as virtual reality.

As children with intellectual disabilities are prompted to perform tasks of VR and computer-based cognitive therapy, they become more familiar with them, which in turn can expand the complexity of the world they perceive around them. Therefore, skills that are typically underdeveloped, such as perceptive visual and motor stimulation, can be made more prominent in targeted VR programs [37]. A previous study hypothesized that practice of these activities could address therapeutic goals, including improved visual-perceptual processing, postural control, and functional mobility [23]. In this study, we identified that the VR intervention appeared to stimulate visual-motor integration. Additionally, the intervention promoted the participants' abilities to use multiple parts of the body together, such as eye-hand coordination and visual-motor speed. These are important elements of a child's developmental process and the basis for further developmental processes. Thus, our findings support a positive impact of VR interventions on child development.

Prior studies have reported on VR training for children with neurodevelopmental disabilities, and improvements have been observed in children with ASD, developmental coordination disorders, and cerebral palsy. However, few studies have been conducted on children with intellectual disabilities. For children with intellectual disabilities, cognitive deficits are a major problem, so it is necessary to perform computer-based cognitive therapy rather than just VR. Results of this study provide useful evidence of the utility of VR and computer game-based cognitive therapy in improving visual-motor integration in children with intellectual disabilities. The major advantage of VR and computer-based cognitive therapy is that the individual knows that the computer environment is not real, but the mind and body behave as if they were real. Therefore, people can face difficult situations in virtual much more easily than in reality and may be open to trying new treatment strategies. Our findings suggest that the use of VR and computer-based cognitive therapy increased visual perception and motor functions.

Visual-motor integration constitutes an integral part of an individual child's psychological education or neuropsychological assessment for a school or rehabilitation plan [11] Previous studies have shown that VR and computer game-based methods can be used to improve access to the most effective psychotherapy methods [38]. There is a limitation in this study that the psychological aspects of children were not evaluated. Another limitation of this study was the small number of participants and no control group. Future studies should include randomized controlled trials involving a larger number of participants. It should also be investigated whether the effectiveness of VR and computer game-based cognitive therapy is maintained over the long term.

## 5. Conclusions

This study found that VR and computer game-based cognitive therapy for visual-motor integration is an effective training method for children with intellectual disabilities that promote visual perception and motor function. Thus, it provides evidence supporting the utility of VR and computer game-based cognitive therapy in children with intellectual disabilities. This study suggests that VR and computer game-based cognitive therapy may be effective for children with intellectual disabilities and needs to be expanded to a randomized controlled design study targeting many participants in the future.

## Figures and Tables

**Table 1:** Characteristics of participants (*N* = 13).

Characteristics		Range (*M* ± SD)	*N* (%)
Gender	Male		7 (53.8)
	Female		6 (46.2)

Dominant hand	Right		13 (100.0)
	Left		0 (0.0)
Chronological age		7 to 13 (9.2 ± 1.7)	
IQ		55 to 85 (63.4 ± 6.2)	

Motor function	Well-below average		8 (61.5)
	Below average		5 (38.5)
	Average		0 (0.0)
	Above average		0 (0.0)
	Well-above average		0 (0.0)

General visual perception	Very poor		4 (30.8)
	Poor		1 (7.7)
	Below average		3 (23.1)
	Average		3 (23.1)
	Above average		2 (15.4)

General visual perception is an index calculated from Developmental Test of Visual Perception—2nd Edition; motor function is an index calculated from Bruininks-Oseretsky Test of Motor Proficiency—2nd Edition.

**Table 2:** Differences in score before and after intervention (*N* = 13).

Assessments		*M* ± SD		*z*	*r*
		Pretest	Posttest		
BOT-2	Total standard score	27.15 ± 7.55	35.23 ± 13.09	-2.949^∗∗^	0.744^∗∗^
	Percentile rank	3.46 ± 4.99	19.61 ± 26.87	-2.366^∗∗^	0.631^∗^

DTVP-2	General visual perception	97.76 ± 23.21	105.92 ± 28.13	-2.750^∗∗^	0.893^∗∗^
	Visual motor integration	86.46 ± 22.42	90.46 ± 27.07	-1.335	0.908^∗∗^
	Eye-hand coordination	87.38 ± 44.61	101.00 ± 50.38	-1.922	0.898^∗∗^
	Copying	16.07 ± 9.57	14.92 ± 8.95	-0.270	0.918^∗∗^
	Spatial relation	13.76 ± 11.29	15.38 ± 12.73	-2.043^∗^	0.920^∗∗^
	Visual motor speed	6.23 ± 4.88	8.84 ± 6.29	-2.203^∗^	0.723^∗∗^
	Motor-reduced visual perception	109.23 ± 23.50	120.61 ± 27.57	-3.066^∗∗^	0.924^∗∗^
	Position in space	11.53 ± 4.53	12.92 ± 6.07	-2.239^∗^	0.942^∗∗^
	Figure ground	12.92 ± 13.04	19.00 ± 15.50	-2.179^∗^	0.871^∗∗^
	Visual closure	5.84 ± 1.57	8.38 ± 4.11	-2.253^∗^	0.537
	Form constancy	9.76 ± 10.76	12.76 ± 11.46	-2.527^∗^	0.798^∗∗^

BOT-2: Bruininks-Oseretsky Test of Motor Proficiency—2nd Edition; CI: confidence interval; DTVP-2: Developmental Test of Visual Perception—2nd Edition. ^∗^*p* < 0.05, ^∗∗^*p* < 0.01; *r*: rank-biserial correlation.

## Data Availability

The data used to support the findings of this study are restricted by the Ethics Committee in order to protect participants' privacy.

## References

[1] American Psychiatric Association (2013). *Diagnostic and Statistical Manual of Mental Disorders (DSM-5®)*.

[2] Schalock R. L., Borthwick-Duffy S. A., Bradley V. J. (2013). *Intellectual Disability: Definition, Classification, and Systems of Supports*.

[3] van der Molen M. J., van Luit J. E. H., Jongmans M. J., van der Molen M. W. (2007). Verbal working memory in children with mild intellectual disabilities. *Journal of Intellectual Disability Research*.

[4] Di Blasi F. D., Elia F., Buono S., Ramakers G. J., Di Nuovo S. F. (2007). Relationships between visual-motor and cognitive abilities in intellectual disabilities. *Perceptual and Motor Skills*.

[5] Woodhouse W., Bailey A., Rutter M., Bolton P., Baird G., Le Couteur A. (1996). Head circumference in autism and other pervasive developmental disorders. *Journal of Child Psychology and Psychiatry*.

[6] Vuijk P. J., Hartman E., Scherder E., Visscher C. (2010). Motor performance of children with mild intellectual disability and borderline intellectual functioning. *Journal of Intellectual Disability Research*.

[7] Boat T. F., Wu J. T. (2015). *Mental Disorders and Disabilities Among Low-Income Children*.

[8] Wuang Y. P., Wang C. C., Huang M. H., Su C. Y. (2008). Profiles and cognitive predictors of motor functions among early school-age children with mild intellectual disabilities. *Journal of Intellectual Disability Research*.

[9] Graf M., Hinton R. N. (1997). Correlations for the developmental visual-motor integration test and the Wechsler Intelligence Scale for Children-III. *Perceptual and Motor Skills*.

[10] Schultz R. T., Carter A. S., Gladstone M. (1998). Visual-motor integration functioning in children with Tourette syndrome. *Neuropsychology*.

[11] Demsky Y., Carone D. A., Burns W. J., Sellers A. (2000). Assessment of visual-motor coordination in 6- to 11-yr.-olds. *Perceptual and Motor Skills*.

[12] Standen P. J., Brown D. J. (2005). Virtual reality in the rehabilitation of people with intellectual disabilities: review. *Cyberpsychology & Behavior*.

[13] Grynszpan O., Nadel J. (2015). An eye-tracking method to reveal the link between gazing patterns and pragmatic abilities in high functioning autism spectrum disorders. *Frontiers in Human Neuroscience*.

[14] Quill K. A. (1997). Instructional considerations for young children with autism: the rationale for visually cued instruction. *Journal of Autism and Developmental Disorders*.

[15] Ayres K., Cihak D. (2010). Computer- and video-based instruction of food-preparation skills: acquisition, generalization, and maintenance. *Intellectual and Developmental Disabilities*.

[16] Grynszpan O., Weiss P. L., Perez-Diaz F., Gal E. (2014). Innovative technology-based interventions for autism spectrum disorders: a meta-analysis. *Autism*.

[17] Kapp K. M. (2012). *The Gamification of Learning and Instruction: Game-Based Methods and Strategies for Training and Education*.

[18] Choi S. J. (2017). *The Effect of Korean Computer-Based Cognitive Rehabilitation Program(CoTras) on the Attention and EEG(Electroencephalogram) Activation for Adults Intellectual Disabilities*.

[19] Rizzo A. S., Kim G. J. (2005). A SWOT analysis of the field of virtual reality rehabilitation and therapy. *Presence: Teleoperators and Virtual Environments*.

[20] Valmaggia L. R., Latif L., Kempton M. J., Rus-Calafell M. (2016). Virtual reality in the psychological treatment for mental health problems: an systematic review of recent evidence. *Psychiatry Research*.

[21] Lorusso M. L., Travellini S., Giorgetti M., Negrini P., Reni G., Biffi E. (2020). Semi-immersive virtual reality as a tool to improve cognitive and social abilities in preschool children. *Applied Sciences*.

[22] Magill R. A., Anderson D. (2010). *Motor Learning and Control*.

[23] Deutsch J. E., Borbely M., Filler J., Huhn K., Guarrera-Bowlby P. (2008). Use of a low-cost, commercially available gaming console (Wii) for rehabilitation of an adolescent with cerebral palsy. *Physical Therapy*.

[24] Esposito M., Ruberto M., Gimigliano F. (2013). Effectiveness and safety of Nintendo Wii Fit PlusTM training in children with migraine without aura: a preliminary study. *Neuropsychiatric Disease and Treatment*.

[25] Kim Y. G., Lee M. J. (2013). The effect on computer-based cognitive rehabilitation program for children (CoTras-C) for the cognitive ability and visual perception in developmental disability. *Journal of Rehabilitation Research*.

[26] Bruininks R. H. (2005). *Bruininks-Oseretsky Test of Motor Proficiency: BOT-2*.

[27] Coallier M., Rouleau N., Bara F., Morin M. F. (2014). Visual-motor skills performance on the Beery-VMI: a study of Canadian kindergarten children. *Open Journal of Occupational Therapy*.

[28] Hammill D. D., Pearson N. A., Voress J. K., Frostig M. (1993). *Developmental Test of Visual Perception: DTVP-2*.

[29] Brown T., Hockey S. C. (2013). The validity and reliability of Developmental Test of Visual Perception-2nd edition (DTVP-2). *Physical & Occupational Therapy in Pediatrics*.

[30] Kerby D. S. (2014). The simple difference formula: an approach to teaching nonparametric correlation. *Comprehensive Psychology*.

[31] Akoglu H. (2018). User's guide to correlation coefficients. *Turkish Journal of Emergency Medicine*.

[32] Schulz M., Reuding T., Ertl T. (1998). Analyzing engineering simulations in a virtual environment. *IEEE Computer Graphics and Applications*.

[33] Memisevic H., Djordjevic M. (2018). Visual-motor integration in children with mild intellectual disability: a meta-analysis. *Perceptual and Motor Skills*.

[34] Murray P. J. (1997). Using virtual focus groups in qualitative research. *International Journal of Qualitative Methods*.

[35] Cromby J. J., Standen P. J., Brown D. J. (1996). The potentials of virtual environments in the education and training of people with learning disabilities. *Journal of Intellectual Disability Research*.

[36] Standen P. J., Brown D. J., Cromby J. J. (2001). The effective use of virtual environments in the education and rehabilitation of students with intellectual disabilities. *British Journal of Educational Technology*.

[37] Standen P. J., Brown D. J. (2006). Virtual reality and its role in removing the barriers that turn cognitive impairments into intellectual disability. *Virtual Reality*.

[38] Freeman D., Reeve S., Robinson A. (2017). Virtual reality in the assessment, understanding, and treatment of mental health disorders. *Psychological Medicine*.

